# *TLE6* mutation causes the earliest known human embryonic lethality

**DOI:** 10.1186/s13059-015-0792-0

**Published:** 2015-11-05

**Authors:** Anas M. Alazami, Salma M. Awad, Serdar Coskun, Saad Al-Hassan, Hadia Hijazi, Firdous M. Abdulwahab, Coralie Poizat, Fowzan S. Alkuraya

**Affiliations:** Department of Genetics, King Faisal Specialist Hospital and Research Center, Riyadh, Saudi Arabia; Cardiovascular Research Program, King Faisal Specialist Hospital and Research Center, Riyadh, Saudi Arabia; Department of Pathology and Laboratory Medicine, King Faisal Specialist Hospital and Research Center, Alfaisal University, Riyadh, Saudi Arabia; Department of Obstetrics and Gynecology, King Faisal Specialist Hospital and Research Center, Riyadh, Saudi Arabia; Department of Anatomy and Cell Biology, College of Medicine, Alfaisal University, Riyadh, Saudi Arabia

**Keywords:** Mendelian, Embryonic lethal, Subcortical maternal complex, Infertility

## Abstract

**Background:**

Embryonic lethality is a recognized phenotypic expression of individual gene mutations in model organisms. However, identifying embryonic lethal genes in humans is challenging, especially when the phenotype is manifested at the preimplantation stage.

**Results:**

In an ongoing effort to exploit the highly consanguineous nature of the Saudi population to catalog recessively acting embryonic lethal genes in humans, we have identified two families with a female-limited infertility phenotype. Using autozygosity mapping and whole exome sequencing, we map this phenotype to a single mutation in *TLE6*, a maternal effect gene that encodes a member of the subcortical maternal complex in mammalian oocytes. Consistent with the published phenotype of mouse *Tle6* mutants, embryos from female patients who are homozygous for the *TLE6* mutation fail to undergo early cleavage, with resulting sterility. The human mutation abrogates TLE6 phosphorylation, a step that is reported to be critical for the PKA-mediated progression of oocyte meiosis II. Furthermore, the *TLE6* mutation impairs its binding to components of the subcortical maternal complex.

**Conclusion:**

In this first report of a human defect in a member of the subcortical maternal subcritical maternal complex, we show that the *TLE6* mutation is gender-specific and leads to the earliest known human embryonic lethality phenotype.

**Electronic supplementary material:**

The online version of this article (doi:10.1186/s13059-015-0792-0) contains supplementary material, which is available to authorized users.

## Background

Thousands of genes are known to exert specific phenotypic effects when mutated individually and are referred to as ‘Mendelian’. The phenotypic consequences of mutations in Mendelian genes involve virtually every known aspect of human physiology and anatomy [1]. Embryonic lethality sometimes represents the severe end of the phenotypic expression of Mendelian genes that are otherwise better known for their postnatal phenotypic consequences [2]. On the other hand, embryonic lethality may be the only phenotypic expression of genes that play a fundamental role in early development [3]. Identifying these genes, therefore, can provide novel insights into basic biological processes during development [1, 4]. The fraction of Mendelian genes in humans that are ‘embryonic lethal’ is unknown but extrapolation from model organisms, for example, 19 % of yeast genes and 30 % of mouse genes are embryonic lethal when knocked out, suggests that they are at least in the hundreds [5, 6].

Consanguineous families facilitate the occurrence of autosomal recessive Mendelian diseases by virtue of rendering founder mutations homozygous through autozygosity, and have greatly aided the discovery of novel Mendelian genes through autozygosity mapping [7, 8]. More recently, combining autozygosity mapping with genomic sequencing has markedly accelerated novel disease gene discovery [9]. Due to practical reasons, the entry phenotypes in these studies is almost always a postnatal recognizable pattern rather than embryonic lethality. We have recently shown that consanguineous families that segregate embryonic lethal phenotypes provide an exceptionally high yield for novel gene discovery where seven novel genes were identified from a study of less than 20 families [3]. However, embryonic lethality in that study was limited to late first trimester and beyond, such that very early embryonic lethal events could not be assessed. In this study, we report the identification of the genetic cause of the earliest known embryonic lethal phenotype through the study of consanguineous families that segregate female-limited infertility and failure of embryonic development beyond the zygote formation.

## Materials and methods

### Human subjects

Families were recruited through the Reproductive Medicine Unit at KFSHRC based on the observation of abnormal embryo development during regular *in vitro* fertilization treatment of consanguineous couples. Eligible families and controls were enrolled after signing a KFSHRC IRB-approved written informed consent (RAC #2121053). Venous blood was collected in EDTA and, when possible, in Na-heparin tubes for DNA extraction and lymphoblastoid cell line establishment, respectively. All methods comply with the Helsinki Declaration.

### Autozygome and linakge analysis

Determination of the entire set of autozygous intervals per genome (autozygome) was through genomewide SNP genotyping (Axiom SNP chip, Affymetrix) followed by mapping of runs of homozygosity as surrogates of autozygosity using AutoSNPa v4, as described before [10, 11]. Overlap in the autozygome of affected individuals was employed as a strategy to determine the critical disease locus. Statistical confirmation of the critical locus was achieved by linkage analysis using easyLINKAGE [12].

### Whole exome sequencing

Exome capture was performed using TruSeq Exome Enrichment kit (Illumina) following the manufacturer’s protocol. Samples were prepared as an Illumina sequencing library, and in the second step, the sequencing libraries were enriched for the desired target using the Illumina Exome Enrichment protocol. The captured libraries were sequenced using Illumina HiSeq 2000 Sequencer. The reads are mapped against UCSC hg19 [13] by BWA ver.0.5.9rc1 [14], without unordered sequences and alternate haplotypes. The Picard-tools suite (ver.1.59) was then utilized to sort by mapping coordinates, and BEDtools (ver. 2.15.0) filtered out any reads not present in the targeted exonic regions. SNPs and Indels were detected by SAMTOOLS ver.0.1.18 [15] and annotated using ANNOVAR ver.Nov 2011 [16]. The candidacy of the resulting variants was based on their physical location within the autozygome of the affected individual, their population frequency and predicted effect on the protein as described before [9]. Data used in this paper come from a small and well-defined family. To protect the identity of individuals, these confidential data are not publicly available.

### Western blot and phosphorylation analysis

Epstein Barr Virus (EBV) transformed cell lines were produced from three healthy donors (controls) and from three individuals who are homozygous for the TLE6 S510Y mutation (patients, see below). Western blot analysis was performed as described [17]. Briefly, cells were harvested by centrifugation and resuspended in lysis buffer (20 mM Tris pH 7.5, 350 mM NaCl, 0.05 % β-mercaptoethanol) supplemented with a protease inhibitor cocktail. After sonication and centrifugation, 30 μg of total cell lysates were analyzed by SDS-PAGE on 10 % acrylamide or on 8 % Phospho-tag acrylamide gels (Wako, TX, USA), followed by transfer of the proteins onto nitrocellulose membrane. After blocking in 5 % milk in TBS-Tween, the membranes were incubated with anti-TLE6, anti-KDHC3L/Ecat1 from (Abcam, Cambridge, MA, USA) or anti-OOEP, anti-Flag and anti-GAPDH from (Santa Cruz, CA, USA). After washing, secondary reactions were carried out with biotin conjugated secondary antibodies followed by anti-avidin-HRP conjugated antibody. Signals were visualized using an LAS 4000 mini (GE Healthcare, UK) and quantified using ImageQuant software (GE Healthcare, UK).

### Phosphatase inhibitor treatment

A total of 30 μg of whole cell lysates from the two control individuals were incubated with 40 μM of calf intestine alkaline phosphatase (CIP) (Promega, Madison, WI, USA) and equimolar amount of PKI (5–24), PKA Inhibitor (Santa Cruz, CA, USA) in a reaction buffer (50 mM Tris–HCl, pH 9.3; 1 mM MgCl2; 0.1 mM ZnCl2; 1 mM spermidine). Extracts were then analyzed by western blot analysis as described before [17].

### Production of TLE6 S510Y protein

Mutant TLE6 S510Y was produced using the Stratagene site-directed mutagenesis system (Stratagene, La Jolla, CA, USA) according to manufacturer’s instructions; using Flag tagged TLE6 plasmid (Origene, Rockville, MD, USA). Primers used to generate the mutation are sense: 5′-TCCTGAGCGTCAAGTTCT(A)CCCCTTTGGCCAGTGGTG-3′ and anti-sense: 5′-AGGACTCGCAGTTCAAGATGGGGAAACCGGTCACCAC-3. The base change was from C to A, shown between brackets. After verification of the mutation by sequencing, control transformed lymphocytes were transfected with wild-type and mutant plasmids using lipofectamine (Invitrogen, Waltham, MA, USA). Twenty-four hours post transfection, total cell lysates were prepared and analyzed by western blotting or immunoprecipitation.

### Immunoprecipitation

Cell extracts were re-suspended in a buffer containing 50 mM Tris pH7.6, 150 mM or 500 mM NaCl, 1 mM EDTA, 1%Triton X-100, 1 mM PMSF supplemented with phosphatase and protease inhibitor cocktail (Sigma). A total of 60 μg of total protein was incubated with Flag-IgG sepharose beads (Sigma-Aldrich, St. Louis, MO, USA) or Protein A dynabeads (Invitrogen, Waltham, MA, USA) coupled to anti-OOEP antibody (Santa Cruz, CA, USA) or control IgG in a pull-down buffer (50 mM HEPES pH 7.5, 1 mM EDTA, 150 mM NaCl, 10 % glycerol, 0.1 % Tween 20, 0.5 mM DTT, 1 mM PMSF, 2 μg/mL leupeptin, and 2 μg/mL pepstatin A). Extracts were incubated with the beads for at least 2 h at 4 °C while mixing on a rotating wheel. After collection of the supernatants, the beads were washed with pull-down buffer and left as a 50 % slurry after a final wash. After elution, proteins were loaded on 10 % SDS-PAGE gel and immunoblotting was performed using the indicated antibodies.

### *In vitro* phosphorylation

Wild-type and TLE6-S510Y proteins were expressed in HEK293 and immunoprecipitated using anti-Flag beads (Sigma-Aldrich, St. Louis, MO, USA). A total of 20 μg of purified proteins was added to a standard PKA mixture containing 20 mM Hepes (pH 7.5), 5 mM MgCl2, 1 mM unlabeled ATP, 1 mM 1,4-dithiothreitol, 100 mM NaCl, and 1 mM [γ^32^P]ATP (0.5 Ci/mmol- Perkin Elmer, Waltham, MA, USA), with different amounts of PKA (Promega, Madison, WI, USA) as indicated and incubated for 1 h at 37 °C. Reactions were stopped by adding 20 μL of 2X Laemmli sample buffer and proteins were separated by SDS-PAGE electrophoresis followed by autoradiography and immunoblotting.

## Results

### Identification of a preimplantation embryonic lethal phenotype

Failure of *in vitro* fertilization (IVF) can be caused by a multitude of factors. However, failure of fertilization despite intracytoplasmic injection of apparently healthy sperms in apparently healthy eggs is highly unusual. In our last 20 years of experience with thousands of IVF cycles, we have observed this pattern consistently only in eight couples, two of whom were consanguineous and available for recontacting. Family 1 consists of two sisters who presented separately for treatment of primary infertility. Each of the sisters had four intra-cytoplasmic sperm injection (ICSI) cycles in another hospital with no fertilization and were therefore referred to our hospital (at ages 26 and 36 years, respectively) where they had a total of five cycles, comprising the successful stimulation and retrieval of 58 oocytes. Only three oocytes developed two pronuclei indicating normal fertilization. These zygotes had developmental arrest at the one-, two-, and four-cell stage. These two sisters have a number of sisters and brothers who are healthy and fertile. Family 2 was also consanguineous and the index presented with her husband at the age of 30 years for treatment of primary infertility. The pattern of IVF failure was similar to that described in Family 1 and two ICSI cycles with total 19 oocytes injected resulted in no zygote formation.Because of the potential for identification pedigrees for these two families are not shown in this paper.

**Fig. 1:**
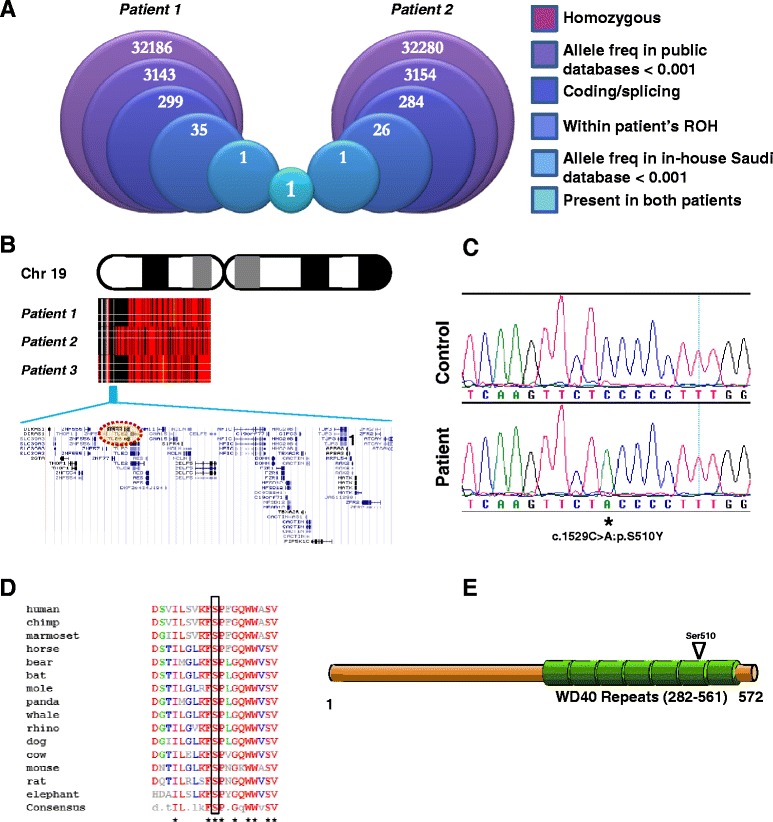
Two families with primary infertility link to a mutation in *TLE6*. **a** The two affected sisters from Family 1 were separately subjected to whole exome sequencing following the indicated pipeline. Each sister was examined based only on her own regions of homozygosity (ROH), and the only surviving variant was found common to both. **b** High density genotyping output for chromosome 19p (using AutoSNPa software [10]), with red indicating heterozygosity and black homozygosity. The three affected women share an identical haplotype which encompasses 42 genes, based on UCSC Human Genome Browser data. **c** DNA chromatogram of the *TLE6* mutation, with a normal control sequence for comparison. **d** TLE6 orthologues appear to be present only in mammals, wherein there is universal conservation of the affected serine residue (boxed). Sequence data were acquired on NCBI-BLAST then collated using Multalin [28]. **e** Serine 510 is located within a cluster of WD40 domain repeats that make up most of the protein’s C-terminal half

### Identification of a novel disease locus for early embryonic lethality defined by TLE6 mutation

Although the phenotype in the three women is primarily failure of zygote formation, the occasional formation of zygotes that did not divide suggested that the phenotype in these patients can be considered preimplantation embryonic lethality. The observation of this phenotype only in certain female members of each family suggests that this phenotype is female-limited, perhaps due to a recessive mutation of a maternal effect gene. To identify the likely causal mutation we performed whole-exome sequencing in two of the three affected women and only considered homozygous, coding/splicing variants within the autozygome of each individual. Only one novel variant remained after applying these filters in both exomes, a homozygous substitution in *TLE6* (transducin-like enhancer of split 6, OMIM ID: 612399; NM_001143986.1:c.1529C > A:p.S510Y) (Fig. 1b, d). Reassuringly, this variant was present in an autozygous interval that is shared by the three females (chr19: 2712016–3918047, hg19) (Fig. 1c). The identical haplotype indicated the presence of a common ancestor, consistent with the shared geographic location of the two families. Furthermore, linkage analysis showed that this haplotype is the only haplotype to achieve genomewide significance with LOD of 4.17 (Additional file 1: Figure S1).

This variant was confirmed by Sanger sequencing to be homozygous in the three female patients. Consistent with this mutation exerting a gender-limited phenotype, we also had access to one fertile brother who was also homozygous for this variant (Fig. 1a). This mutation was only observed once in the heterozygous state in 615 in-house Saudi exomes (allele frequency <0.001) and was absent in the 1000 Genomes and Exome Variant Server databases. The affected residue appears to be universally conserved among mammalian TLE6 orthologs (Fig. 1e), and is present within a stretch of seven WD40 domain repeats, which dominate the protein’s C-terminus (Fig. 1f).

### TLE6 mutation causes impaired phosphorylation

**Fig. 2:**
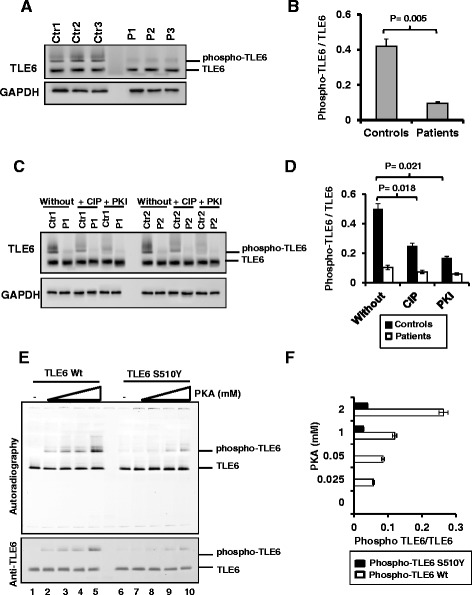
Abrogation of TLE6 protein phosphorylation due to TLE6 missense mutation. **a** Western blot using phospho-tag acrylamide gels showing a shift of phosphorylated TLE6 and significant reduction of phosphorylated TLE6 in patient lymphocytes compared to normal controls. GAPDH is shown as loading control. **b** Quantification of A. **c** Phosphorylation of TLE6 is reduced in control lymphocytes treated with 40 μM calf intestine phosphatase (CIP) and PKA inhibitor (PKI). **d** Quantification of C from two biological repeats and two technical repeats. **e** *In vitro* phosphorylation of wild-type and S510Y TLE6 by PKA in the presence of [γ32P] followed by autoradiography and immunoblotting using anti-TLE6. **f** Quantification of E from three independent repeats. Ctr: Control cells, P: Patient cells

The variant we identified in *TLE6* is predicted to be pathogenic by both PolyPhen (0.991) and SIFT (0) (based on the Ensembl Variant Effect Predictor GRCh37, default parameters), indicating its strong conservation across species and the deleterious calculated effect of the given amino acid substitution. Since TLE6 is known to be phosphorylated by protein kinase A (PKA) [18] and since the mutation replaces a serine residue, we hypothesized that the missense mutation exerts its pathogenicity by disturbing a potential phosphorylation site on TLE6. Immunoblot analysis using three patient-derived lymphoblastoid cell lines, showed a single band instead of the doublet pattern indicative of unphosphorylated and phosphorylated forms of TLE6 that we observed in controls [18] (Fig. 2a, b). Analysis of the extracts using phospho affinity gels [19] enhanced the mobility shift of the phosphorylated form of TLE6, which was detected in control cells whereas phosphorylated TLE6 was markedly reduced (>90 %) in the patient lymphoblastoid cells (Fig. 2a, b). To validate that the slower migrating band corresponded to phosphorylated TLE6, we treated controls and patients extracts with calf intestine alkaline phosphatase (CIP), a generic phosphatase enzyme, and with the PKA specific inhibitor (PKI), and analyzed TLE6 on a phosphor-tag gel. We detected a significant reduction of the slow-migrating form of TLE6 after CIP and PKI treatment of control cells (Fig. 2c, d). Patient cells expressed very low levels of phosphorylated-TLE6, and both CIP and PKI treatment minimally affected TLE6 phosphorylation, showing the specificity of the de-phosphorylation assay (Fig. 2c, d). The residual signals likely correspond to TLE6 phosphorylation by other kinases. Together these results show impaired TLE6 phosphorylation in cells expressing the *TLE6* mutation and indicate the role of PKA in regulating TLE6 phosphorylation.

To confirm that PKA significantly phosphorylates the S510 site in TLE6, we performed *in vitro* kinase assays with increasing concentrations of purified PKA, and wild-type or mutant TLE6-S510Y in the presence of [γ^32^P] ATP. PKA successfully phosphorylated wild-type TLE6 (Fig. 2e, lanes 2–5) while mutant TLE6-S510Y had a significantly lower phosphorylation (Fig. 2e, lanes 7–10). The reduced phosphorylation of TLE6-S510Y by PKA was also observed by immunoblotting (Fig. 2e, lower panel). Thus, these results indicate that the TLE6 mutation at S510 impairs its phosphorylation by PKA.

### TLE6 mutation affects binding to SCMC proteins

**Fig. 3:**
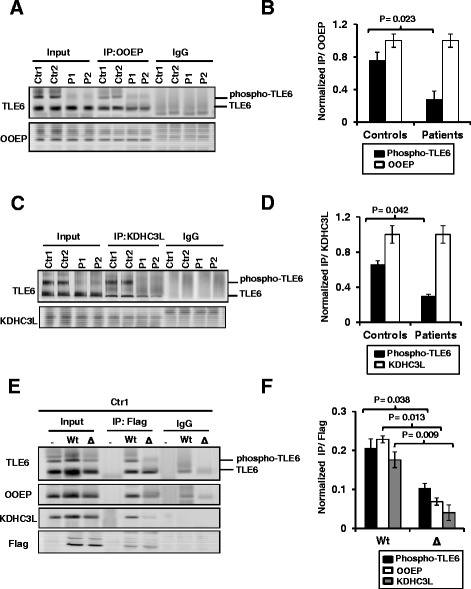
The TLE6 mutation diminishes SCMC protein binding and complex formation. **a** Interaction of endogenous OOEP protein with TLE6 from control and patients cells after immunoprecipitation with anti-OOEP and IgG antibody followed by immunoblotting. **b** Quantification of A from two biological repeats and two technical repeats. **c** Interaction of endogenous KDHC3L protein with TLE6 from control and patient cells after immunoprecipitation with anti-KDHC3L/Ecat1 and IgG antibody followed by immunoblotting. **d** Quantification of C from two independent experiments. **e** Immunoprecipitation performed from HEK293 cells expressing wild-type or mutant TLE6 S510Y with anti-Flag beads followed by immunoblotting, showing reduced binding of OOEP and KDHC3L to mutant TLE6. **f** Quantification of E from two independent repeats

We sought to assess the effect of the TLE6 mutation on binding to SCMC proteins as this may provide a potential mechanism for the observed infertility phenotype [20]. Since clinical materials from unsuccessful ICSIs were unavailable, we tested the effect of the S510Y mutation on the physical binding between TLE6 and two members of the human SCMC (OOEP and KDHC3L). We performed immunoprecipitation and western blot analysis using patient-derived lymphoblastoid cells (endogenous interaction) as well as through transfection experiments (interaction between overexpressed proteins). Patient cells showed a reduced interaction between endogenous OOEP and TLE6 (Fig. 3a, b). Interestingly, the interaction with phosphorylated TLE6 was mostly diminished. Similarly, a reduced interaction between KDHC3L and phospho-TLE6 was also observed (Fig. 3c, d). To investigate whether phosphorylation of S510 is required for TLE6 binding of SCMC members, we over-expressed wild-type or mutant TLE6 S510Y in cells and assessed the ratio of phosphorylated and total TLE6 after immunoprecipitation. Decreased binding of OOEP and KDHC3L to mutant TLE6-S510Y was observed (Fig. 3e, f) indicating that the TLE6 mutation affects the interaction between the SCMC components.

## Discussion

Maternal genes play a critical role in the very early stages of embryonic development because of the lag in transcribing genes derived from the male pronucleus. Although maternal effect genes are very well studied in other model organisms, little is known about them in mammalian oocytes due to practical limitations [21]. Recently, TLE6 was identified as an essential member of the four-protein, murine SCMC along with MATER, Filia, and FLOPED [22]. Mice that are knocked out for *Tle6* exhibit complete infertility that is female-specific, which places *TLE6* as one of the less than 30 maternal effect genes identified to date in mammals [23]. In sheep, expression of the four homologous proteins was recently linked to oocyte development potential [24].

The phenotypic overlap between our patients with *TLE6* mutation and the corresponding mouse model is noteworthy. Ovulation proceeds normally in *Tle6* mutants and the retrieved oocytes appear normal just as we observed in our three patients. The infertility phenotype in *Tle6* mutants specifically involves the zygote stage and beyond. While a comparable number of one-cell zygotes was retrieved from *Tle6* mutant females following hormonal stimulation and natural mating, there was a protracted delay in undergoing the first cleavage and the few that did, succumbed to disintegration and never formed morula, a phenotype similar to that observed in the few zygotes from our patients with homozygous *TLE6* mutation. On the other hand, we note that zygote formation, which appears to have occurred normally in the mouse model, was severely impaired in the human patients. The human homologues of the four SCMC proteins (NLRP5, OOEP, KHDC3L and TLE6) share 39–46 % identity with their mouse counterparts [20], so the mechanism for this difference may lie in interspecies divergence. Nonetheless, these observations suggest that the phenotype is not caused by an impaired receptor mediated sperm-egg physical interaction as shown for Juno mutants but rather a maternal gene that exerts its effect after the introduction of the sperm [25].

The mechanism through which TLE6 exerts its maternal effect is not completely understood. *Tle6*^−/−^ oocytes lack the normal subcortical distribution of the SCMC despite normal abundance of RNA from their component genes, which clearly shows an essential role for TLE6 in stabilizing this complex [23]. Indeed, the *Tle6* mutant phenotype is very similar to that observed in knockouts of the other SCMC components indicating that stabilized SCMC is necessary for the very early stages of embryonic development [22, 26, 27]. Interestingly, this early developmental role of *TLE6* was independently discovered through a study that examined the dynamic function of PKA in meiotic maturation of mammalian oocytes [18]. Suppressing PKA resulted in oocytes that were blocked in meiosis II and TLE6 was found to be a major substrate of PKA in this process. TLE6 phosphorylation occurs in a very narrow window within meiosis II and when this was blocked, oocytes failed to mature.

Our data, based on patient cells as well as the mutant Flag-TLE6 plasmid, demonstrate that the S510Y mutation results in markedly reduced phosphorylation of TLE6 and that this phosphorylation is mostly catalyzed by PKA. Mutant TLE6 also exhibits impaired binding ability to at least two SCMC component proteins. Taken together, these data reveal two, potentially related, phenotypic mechanisms: reduced potential for PKA-catalyzed phosphorylation and reduced potential to bind SCMC. Although it is unclear whether the patient phenotype is due to the absence of phosphorylation of S510, or its substitution with tyrosine, we note that both PKA-catalyzed phosphorylation and binding to SCMC have been proposed in the literature to explain the critical role of maternal TLE6 in supporting peri-implantation embryogenesis [18, 20, 23]. Since serine is a canonical phosphorylation site, it is difficult to separate the two roles and, in fact, our data suggest they are related.

## Conclusions

To the best of our knowledge, this is the first reported mutation in an SCMC gene in humans. Our data suggest that TLE6 mutations are a rare cause of human female-limited infertility and represent the earliest known human embryonic lethality that is explicable by a single gene mutation. Our ongoing analysis of embryonic lethal phenotypes is likely to reveal additional novel genes and contribute to the understanding of the fundamental biological processes that control early human development.

## Additional file

Additional file 1: Figure S1. TLE6 is linked to a novel female-sterility phenotype in humans. Genomewide linkage analysis using all available family members from both study families shows that the only significant linkage peak, that is, LOD >3 is the one corresponding to the founder haplotype spanning *TLE6* on chromosome 19. (PDF 44 kb)
