# Supplementary figures and images for: TLE6 mutation causes the earliest known human embryonic lethality
Source: Genome Biol. 2015 Nov 5;16:240. doi: 10.1186/s13059-015-0792-0 (PMC4634911; doi:10.1186/s13059-015-0792-0)

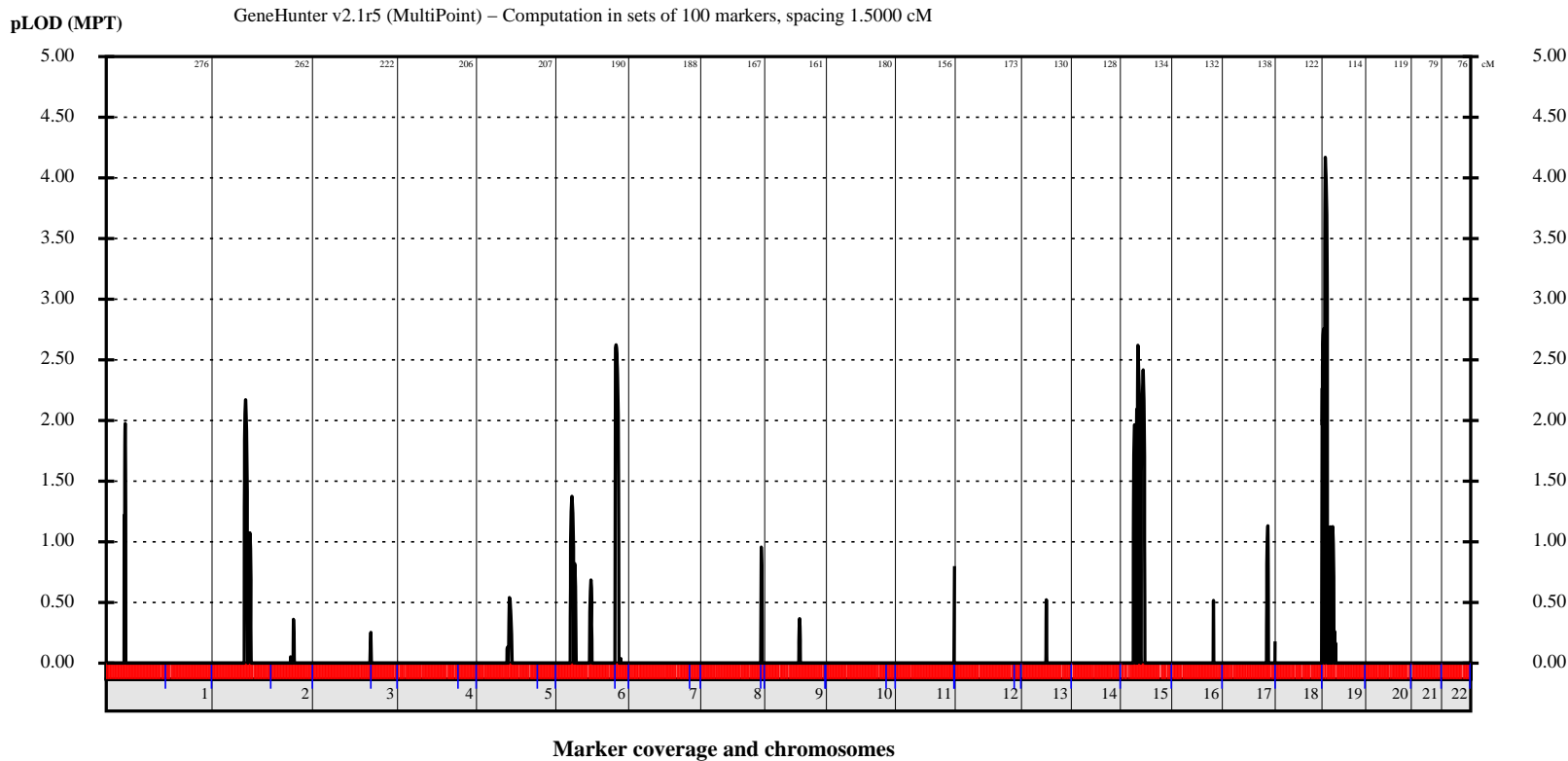

Supplement: Additional file 1: Figure S1. — TLE6 is linked to a novel female-sterility phenotype in humans. Genomewide linkage analysis using all available family members from both study families shows that the only significant linkage peak, that is, LOD >3 is the one corresponding to the founder haplotype spanning TLE6 on chromosome 19. (PDF 44 kb) [file 13059_2015_792_MOESM1_ESM.pdf]
